# The intensive terahertz electroluminescence induced by Bloch oscillations in SiC natural superlattices

**DOI:** 10.1186/1556-276X-7-560

**Published:** 2012-10-09

**Authors:** Vladimir Sankin, Alexandr Andrianov, Alexey Petrov, Alexey Zakhar‘in, Ala Lepneva, Pavel Shkrebiy

**Affiliations:** 1 A.F. Ioffe Physico-Technical Institute, 26 Politekhnicheskaya, St. Petersburg, 194021, Russia

**Keywords:** Terahertz emission, Natural superlattice, Bloch oscillations, Wannier-Stark localization, Transport in high electrical fields, 78.66 Fd; 73.20.Dx

## Abstract

We report on efficient terahertz (THz) emission from high-electric-field-biased SiC structures with a natural superlattice at liquid helium temperatures. The emission spectrum demonstrates a single line, the maximum of which shifts linearly with increases in bias field. We attribute this emission to steady-state Bloch oscillations of electrons in the SiC natural superlattice. The properties of the THz emission agree fairly with the parameters of the Bloch oscillator regime, which have been proven by high-field electron transport studies of SiC structures with natural superlattices.

## Background

The possibility of oscillating motion of electrons in crystals at high field bias has attracted great interest since it was predicted [1,2]. The Bloch oscillations (BO) with frequency (*ν*)

(1) ν=eFa/h,

where *e*, *h*, and *a* are the electron charge, the Plank constant, and the crystal lattice period, respectively, originate from the acceleration of electrons in an electric field and their Bragg reflection at the Brillouin zone boundary. One important condition must be satisfied to achieve the BO regime, namely:

(2) $$\text{eFl}\geq E_{1}\;\text{or}\;F\geq F_{t}=\frac{E_{1}}{\text{el}}$$

where *l* is the electron mean free path, *E*_1_ is the width of the allowed electron band (the subscript 1 corresponds to the bottom electron band), and *F*_*t*_ is the threshold electric field of the BO regime. A thorough analysis of the electron localization effect in high electric fields [3] has revealed that the energy continuum splits into the discrete, so-called Wannier-Stark ladder states in the electric field, which are the frequency-domain equivalent of Bloch oscillations. Esaki and Tsu, in their pioneering work on semiconductor superlattices [4], pointed out that BO of electrons in superlattices with narrow minibands can be observable even for modest fields. Photocurrent, photoluminescence, and electroreflectance experiments on biased structures with artificial superlattices of GaAs/AlGaAs [5,6] have proven the existence of the Wannier-Stark localization (WSL) effect in semiconductor superlattices. The most substantial evidence for BO in artificial superlattices was found in experiments with ultrashort light pulses [7-12]. The results of [7-12] demonstrated a few cycles of BO, which, however, were damped after 1 to 2 ps. It was suggested [13] that the fast damping of BO in artificial superlattices originates from electron scattering at the interfaces of heterojunctions and also from breaking the equidistance of the Wannier-Stark levels. The fast decay of BO leaves room for doubts about the possibility of the practical application of this phenomenon [13]. Apparently, the present level of semiconductor technology cannot provide artificial superlattices with sufficient quality suitable for the creation of terahertz (THz) emitters based on BO.

One interesting system demonstrating superperiodicity and potentially having none of the aforementioned drawbacks is a natural superlattice (NSL) in SiC crystals. It is known [14,15] that all SiC polytypes, excluding the cubic 3C-SiC and hexagonal 2H-SiC, exhibit superperiodicity in the direction along the crystal c-axis in a similar way to crystals with artificial superlattices. The superperiodicity is absolutely stable and has precise crystalline parameters [14]. This superperiodicity is self-organized in the main SiC crystal lattice, and the NSL periods, d, for such polytypes as 4H-, 6H-, and 8H-SiC are equal to 5, 7.5, and 10 Å, respectively [14]. The elementary cell of the hexagonal polytype 4H-, 6H-, and 8H-SiC contains 8, 12, and 16 atoms, which is many times higher than the number of atoms in the 2H-SiC polytype elementary cell. Therefore, in accordance with the theory of Brillouin Zones (BZ) [16], the electron spectrum, for example, of the 6H-SiC crystal in the direction of the c-crystal axis should be considered in the extended BZ composed of six classical Brillouin zones of the crystal. In this case, the electron energy is not a continuous function of wave vector but undergoes breakups at certain planes in k-space. It was shown that the major energy breakups occur at 2*Π*/*c* and 4*Π*/*c* points for 6H-SiC [15], and at 2*Π*/*c*, 4*Π*/*c*, and 6*Π*/*c* points for 8H-SiC, where c is the size of the elementary cell along the c-axis. It explains the appearance of the NSL with the period *d *=* c*/2 and the miniband electron spectrum in hexagonal SiC polytypes.

Electron transport studies on SiC structures with the natural superlattices [17,18] have demonstrated pronounced effects of the negative differential conductivity (NDC) caused by the Wannier-Stark localization phenomenon. The threshold fields of the NDC onset at 300 K were found to be *F*_*t*_ ≈ 110 ± 25 kV/cm, *F*_*t*_ ≈ 150 ± 30 kV/cm and *F*_*t*_ ≈ 290 ± 60 kV/cm for the 8H, 6H, and 4H polytypes, respectively [18], which is in good agreement with Equation 2. It is important to note that the observed values of the NDC [18] were two orders of magnitude higher compared with that reported for artificial superlattices (see, for example, [19-21]). These results mean that there is a well-grounded hope of achieving THz emission due to the Bloch oscillations in SiC NSL. This paper reports on the experimental observation and studies of THz electroluminescence (1.5- to 2-THz spectral range) from SiC structures with a natural superlattice. We attribute this emission to the electron Bloch oscillations in the SiC natural superlattice.

## Methods

### Theory of electron transitions in the Wannier-Stark ladder of silicon carbide natural superlattices

**Figure 1:**
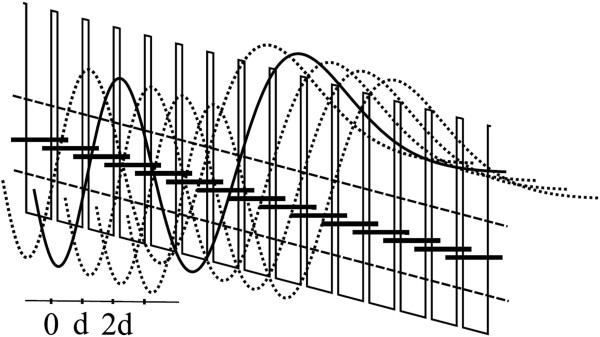
**Schematic diagram of Wannier-Stark states.** Schematic diagram of Wannier-Stark states in a 6H-SiC natural superlattice at a modest uniform electric field. The solid and dotted curves depict the envelope wave functions corresponding to different steps in the ladder. The thick short lines correspond to the electron Wannier-Stark levels confined within the first well (dashed lines).

The internal quantum yield of the THz emission due to optical transitions between Wannier-Stark levels in the natural superlattice of 6H-SiC was estimated. The band diagram of the 6H-SiC NSL depicted in Figure 1 was used for these estimations.

The values for the quantum well width and the energy width of the first electron miniband were taken to be consistent with the experimental data [17,18,22,23] on high-field transport in SiC natural superlattices, which have demonstrated the evolution of the fundamental stages of WSL in these systems (the transition from a relatively small field regime of Bloch oscillations to large field regimes: the Stark-phonon resonances between Wannier-Stark ladder levels, the full localization of the lowermost miniband, and inter-miniband resonance transitions between the first and second minibands). The most important results of these transport studies are summarized in Table 1.

**Table 1:** Parameters of minizone transport in silicon carbide polytypes

**SiC poly-type**	***F***_***t***_**for the Bloch oscillation of electrons, (10**^**5**^**V/cm)**	***F***_***t***_**for the Stark-phonon resonance, (10**^**6**^**V/cm)**	***F***_***t***_**for complete localization of the first electron miniband, (10**^**6**^**V/cm)**	***F***_***t***_**for resonance tunneling of electrons between the first and second (10**^**6**^**V/cm)**	**The width of the first electron miniband *E***_**1**_**(meV)**	**The gap between the first and second minibands *E***_**1,2**_**(meV)**	**Saturated velocity *V***_***s***_**of electrons in the first miniband *F*∥*C*,(*cm*/*s*)**
4H	2.9 [18]	1.6,					
		2.0 [22]			≈ 500		3.3 × 10^6^[24]
6H	1.5 [18]	0.6, 1.1,					
		1.37 [22]	1.8 [22]	1.9 [22]	260 [22]	176 [22]	2.0 × 10^6^[24]
8H	1.1 [18]				≈ 140		1.0 × 10^6^[24]
15R							1.2 × 10^6^[24]
21R							4.4 × 10^3^[23]

Parameters of minizone transport in silicon carbide polytypes. Here, *F*_*t*_ is the threshold field, and (Est.) means an approximate estimation made on the basis of the experimental value of *E*_1_ for the 6H-SiC polytype and taking into account the fact that $E_{1}\propto k_{d}^{2}$, where *k*_*d *_=* ħ*/*d *and d is the NSL period. *F*, electric field. *F*_*t*_, threshold electric field.

Using the tight-binding approximation developed for such systems by Bouchard and Luban [25], the amplitude of the electron wave function can be expressed by the transfer integrals between the nearest quantum wells *V*_1_:

(3) $$|n\rangle =\overset{\infty }{\underset{l=-\infty }{\sum }}J_{l-n}\left(\frac{2V_{1}}{\text{eFd}}\right)u_{l}(z),$$

where *J*_*n*_ is the Bessel function of the first kind, order *n*, and *u*_*l*_(*z*) is the wave function of the electron with the energy *E*_*l *_=* leFd *in the ground state of the quantum well. Taking into account Equation 3, it is straightforward to calculate the probability of an optical transition of an electron between two adjacent quantum wells:

(4) $$\tau_{\text{rad}}^{-1}=\frac{4e^{2}\hbar \omega }{3\Pi^{2}d^{2}{\left(c/n\right)}^{3}m^{2}},$$

where *c*/*n* is the speed of light in SiC, *ℏω *=* eFd *is the energy of the emitted quantum, and *m* is the electron effective mass. We suppose that the electric field is insufficient for the inter-miniband resonant tunneling of the electrons, and the major nonradiative process is the transition between the nearest Wannier-Stark states with the emission of the longwave acoustic phonons. The corresponding probability of the nonradiative process is

(5) $$\tau_{\text{nonrad}}^{-1}=\frac{\Xi^{2}\sqrt{2m}{\left(\text{eFd}\right)}^{5/2}}{\Pi^{3}d\hbar^{3}s^{3}\rho V_{1}},$$

where Ξ is the deformation potential constant, *s* is the speed of sound, and *ρ *is the SiC material density. The formula (5) is applicable only for a low electric field, which satisfies $\text{eFd}\ll \frac{2\Pi s\hbar }{d}$. There are two factors determining the strong dependence of $\tau_{\text{nonrad}}^{-1}$ on the electric field. Firstly, the conservation laws limit the phonon wave vectors to small values in the weak electric fields. Secondly, the electron wave function involves many quantum wells, in this work ( $\frac{E_{1}}{\text{eFd}}\approx 40$), and the contributions *u*_*l*_(*z*) from different quantum wells partly compensate each other. The internal quantum yield, *η*, of the THz emission is

(6) $$\eta =\frac{L}{d}\times \frac{\tau_{\text{rad}}^{-1}}{\tau_{\text{nonrad}}^{-1}}$$

where *L* is the NSL thickness.

### Experimental content

**Figure 2:**
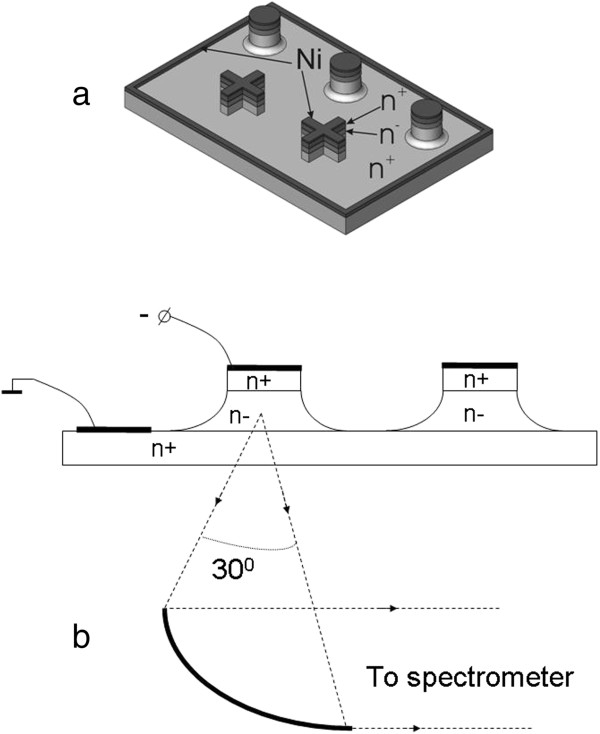
**General view of the structures. (a)** General view of the structures used for THz measurements. **(b)** Geometry of the experiment.

The samples studied in this work were unipolar 6H-SiC *n*^ + ^−* n*^− ^−* n*^ + ^ diode structures. The *n*^−^ epitaxial layer (the base of the diode) was grown by the sublimation on-axis method [26] on a 6H-SiC (0001) Lely substrate, which had *N*_*d *_−* N*_*a *_≈ 2 × 10^18^cm^−3^ and a thickness of 200 *μ*m. The base had a donor concentration of 10^15^cm^−3^ <* N*_*d *_−* N*_*a *_< 10^16^cm^−3^, and the thickness was varied in the interval of 2 to 4 *μ*m. The 6H-SiC polytype of the epitaxial layer and its acceptable doping homogeneity were confirmed by X-ray diffraction, photoluminescence microscopy, and C-V measurements. The latter measurements were done using auxiliary Schottky barriers created on the *n*^−^ layer surface. The top *n*^ + ^layer with *N*_*d*_−*N*_*a *_≈ 10^20^cm^−3^ was fabricated on the *n*^−^ epitaxial layer by ion implantation of nitrogen with subsequent annealing. Finally, the cylindrical mesa-structures with a diameter of 50*μ*m (*S *= 2.0 × 10^−5^cm^2^) and cruciform mesa-structures (*S *= 3 × 10^−5^cm^2^) (Figure 2a) were made by dry etching after photolithography. For a contact, a sputtered and annealed (900°C) nickel film with a thickness of 0.2 *μ*m was used. A common contact area was located on the upper surface of the substrate. Insulator layers were created by proton implantation on the mesa-periphery and on the upper surface of the substrate. In accordance with the aforementioned property of the 6H polytype of SiC crystals, such a diode structure was a natural supperlattice as a whole.

The samples suitable for THz electroluminescence experiments were chosen from a number of the prepared mesa-structures by means of analyzing their *I-V * characteristics at 300 K. The criterion for making this choice was the observation of a mobile high-field domain, which thereby confirmed the development of the WSL effect in the mesa-structure [27]. The small mesa size contributed to a higher yield of high-quality structures. The diode structures selected in this way were used for investigations of the terahertz emission at liquid helium temperatures. Low temperatures are preferable for this kind of experiments because the intensity of electron scattering is reduced. Furthermore, the very low carrier density at helium temperatures and the small thickness of the *n*^−^ layer contributed to a reduction in the probability of domain formation.

For the low-temperature experiments, the samples were placed on an insulating p-type SiC base. After silver wire bonding, the assembly was mounted on the cold finger of an optical cryostat, which was optimized for the THz spectral domain. The geometry of the available structures only permitted the observation of THz radiation though the substrate. Parabolic mirror optics were used to collect the THz emission in the direction normal to the substrate surface within a solid angle of ≈30° (Figure 2b). Polarization of the emission was not expected in this geometry of the experiment, and therefore, all measurements were made in the regime of integrated polarization.

The sample under test was fed with a train of eight pulses, where each pulse in the train was 1 *μ*s in duration with a 950-*μ*s time interval between the pulses. It was paused in 7 *μ*s, after which the next train was begun. Thus, the repetition rate was about 75 Hz. Such a bias was used to minimize lattice heating effects. The duty cycle of the train was 50% at a frequency of 75 Hz. Such a bias was used to minimize lattice heating effects. The spectral measurements were performed with a spectral resolution of 0.6 meV using a Fourier spectrometer operating in the step-scan mode described elsewhere [28]. To eliminate any influence of water vapor absorption, the internal volume of the spectrometer was evacuated down to a residual pressure level of 6 × 10^−2 ^Torr. The THz emission signal was measured using a liquid-helium-cooled silicon bolometer and a lock-in amplifier.

## Results and discussion

**Figure 3:**
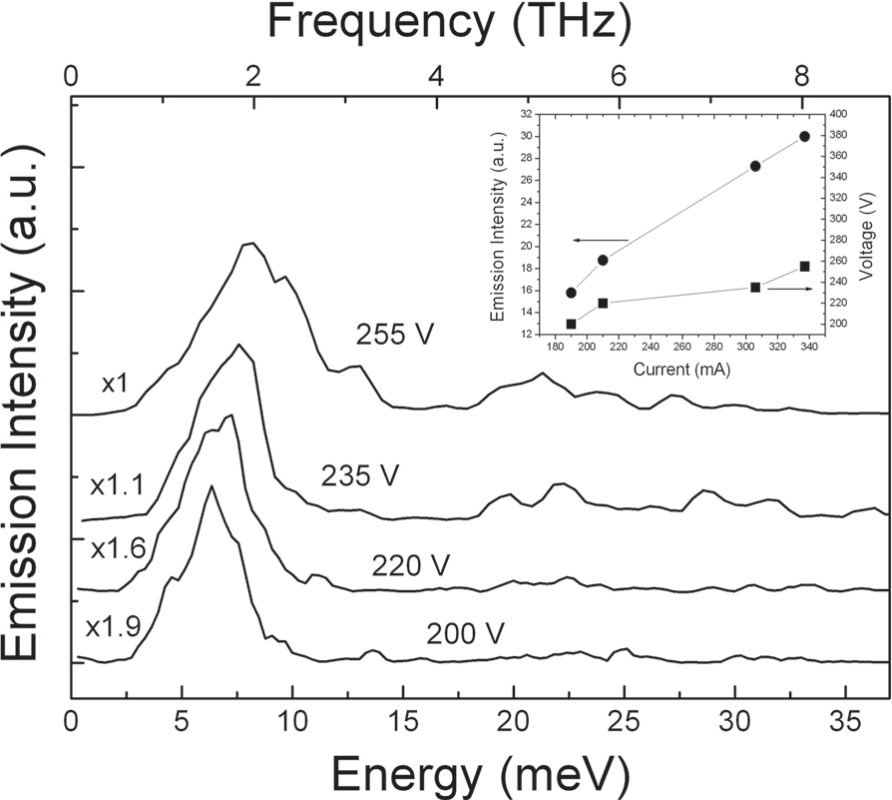
**Spectra of the THz emission.** Spectra of the THz emission from the cruciform SiC mesa-structure at several bias voltages. *T *≈ 7 K. The spectra werre corrected for the spectral response of the measurement system, normalized to the emission maximum, and vertically shifted for clarity. The scaling factors are shown in the graph. The figure insert demonstrates the dependencies of the THz emission intensity and the voltage drop on the current through the structure.

An intensive THz signal was detected at bias voltages exceeding 190 to 195 V. The existence of such a threshold voltage for the THz emission can be explained by the impurity breakdown in the top *n*^ + ^-SiC layer, which is required for injection of electrons into the NSL. Upon reaching the breakdown of the top layer of the structure, the bias voltage becomes redistributed, and some part of it starts to drop to the base of the structure (*n*^−^ layer). At the same time, the intensity of the THz emission begins to grow almost linearly with increases in current. The *I-V * characteristic of the 6H-SiC *n*^ + ^−*n*^−^−*n*^ + ^diode structures and the dependence of the intensity of THz emission on the current are demonstrated in Figure 3 (insert). The current is practically absent at voltages below ≈180 V due to extremely small carrier concentration in the active region of the structure at helium temperatures, and the current appears only as a result of the electron injection from the top *n*^ + ^layer.

Taking into account the precise calibration of the detector used here with the emission of a black body source and also the measured attenuation factor of the experimental instrumentation, we can conclude that the spectrally integrated THz emission peak power for the SiC mesa-structure is about 10*μ*W at 46.2 W of peak pumping power (0.21 A, 220 V). The corresponding external quantum yield of the THz emission is about 0.01 THz photons/electron. Estimations of the internal quantum yield ( Equation 6) of the THz emission for the 6H-SiC structures studied here with a NSL thickness of 2*μ*m result in a value of $\eta =\frac{L}{d}\times \tau_{\text{rad}}^{-1}/\tau_{\text{nonrad}}^{-1}\approx 2,667\times (1.8\times 10^{5}\;s^{-1}/1.3\;\times 10^{10}\;s^{-1})\approx 0.04$. This value is in reasonable agreement with the experiment if the non-optimality of the experimental geometry is taken into account.

In Figure 3, a set of THz electroluminescence spectra measured at different amplitudes of the bias voltage are demonstrated. It is seen that the THz emission spectrum consists practically of a single line, the maximum of which evidently shifts to higher frequencies with increases in the bias voltage. The shift in the emission maximum is in the order of 1.5 meV as the bias is varied from 200 to 255 V. The spectrally integrated THz emission power is about 26 *μ*W for the amplitude of the bias voltage of 255 V (see Figure 3).

**Figure 4:**
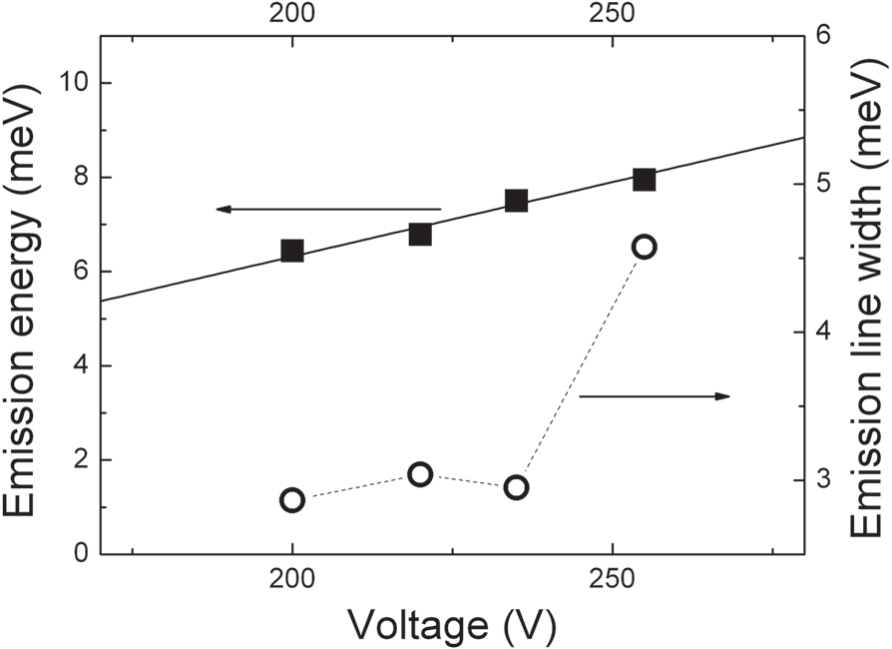
**Dependencies of the peak position on the bias voltage.** Dependences of the peak position of the THz emission line and the emission line width on the bias voltage. The solid line corresponds to a linear fit of the experimental emission maximum (*E*_max_) versus bias voltage (*V *) with a gradient of ≈32 *μ*eV/V. The dashed line is a guide for the eye.

As seen from Figure 4, the shift of the THz emission peak versus the bias voltage can be well approximated by linear law with a gradient of ≈32*μ*eV/V. It is important to note that the FWHM of the emission line is almost constant and equal to ≈2.9 meV (0.7 THz) as the bias voltage varies from 200 to ≈240 V (Figure 3). The shape of the emission spectrum at high bias voltages (255 V and higher) is caused by the superposition of two emission bands. The first of them pertains to the above mentioned series of Bloch emissions at about 6 to 7 meV. The maximum of the second emission band is at about 13 meV. The manifestation of this band can be seen on spectral line at 255 V voltages. We tentatively attribute this band at ≈13 meV to the optical transition of electrons over the next Wannier-Stark ladder state in the high-field-biased NSL of SiC. We have to note that a more detailed theoretical analysis is required for precise interpretation of the experimental findings. At this moment, it is important to underline that similar spectra have been observed for 4H- and 8H-SiC NSL structures, and its spectral features correlate with aforementioned miniband widths and periods of NSL in 4H-, 6H-, and 8H-SiC.

The experimental data allow the observed THz electroluminescence to be attributed to spontaneous radiation resulting from electron Bloch oscillations in a SiC natural superlattice. Using Equation 1 and the fact of the linear dependence of the emission peak position on the bias voltage for the 6H-SiC structures with NSL, the electric field strength *F* required for the BO can be estimated. The estimations give *F* in the order of 8.5 × 10^4^V/cm (at 200 V), which is slightly less than the magnitude of *F*_*t *_for the BO regime obtained from high-field transport measurement data at 300 K on 6H-SiC (see above). However, the agreement between the electric fields is quite reasonable if an increase of the electron scattering time at 7 K compared to its value at 300 K and a corresponding decrease of the threshold field of the BO regime are taken into account. The value of the electric field *F* implies that only a small part (less than 10%) of the bias voltage drops on the base of the structure. The main voltage drops are on the top *n*^ + ^ layer for supporting of the impurity breakdown, on the substrate, and also partly on the contact regions. Nevertheless, the voltage drop on the *n*^−^layer (and hence the electric field *F*) is proportional to the total bias voltage, and this causes the observed linear dependence of the emission peak on the bias (Figure 4). It is necessary to add that the experimental geometry used (Figure 2b) was far from optimal since only a small fraction of the THz emission, mainly propagating along the substrate plane, can be collected in this configuration. Therefore, in the case of an optimal geometry (i.e., observation from the side facet of the structure or from the top and using a diffraction grating deposited on the *n*^ + ^ layer), the expected external quantum yield of the THz emission should be higher by some times.

It necessary to point out that Equation 5 also describes the *I-V * characteristic of the active region of the structure in a regime when the Wannier-Stark ladder states are well formed, and the electron transport is controlled by nonradiative transitions of electrons between the Wannier-Stark levels. In this case, the *I-V * characteristic should obey the *I*∝*F*^5/2^ dependence and not have a region of NDC. The sharp increase of the current (see Figure 1) through the structure under the test excludes the existence of high field domains in the structure in the region of the temperatures and electric fields used in our experiment.

We have measured the polarization properties of the THz emission on specially designed *n*^ + ^−*n*^−^−*n*^ + ^structures allowing for the emission observation from a side facet. It was found that the emission is linearly polarized along the c-crystal axis (along the electric field), and the polarization degree attains 50% at least.

## Conclusions

The previous results on the observation of Wannier-Stark quantization of electrons in silicon carbide NSLs obtained from transport experiments have given a hope of obtaining THz emission in this system. The new experimental data serve as evidence of the existence of steady-state THz radiation induced by Bloch oscillations. The intensive THz emission in the spectral range 1.5 to 2 THz has been found out. The previous papers [13,14,16-18] reported only three to four Bloch oscillation cycles, which were observed using ultrashort laser pulse excitations in different artificial superlattices, where the oscillations were damped after a few picoseconds. The properties of a NSL in SiC crystals allow study-state Bloch oscillations to be achieved under purely electrical excitation. In our opinion, the key factors enabling the achievement of the steady-state Bloch oscillations are the absence of heterointerfaces in SiC NSL and the possibility of electron transport along the sufficiently broad first miniband.

To summarize, THz emission due to electron Bloch oscillations in the steady-state regime has been observed for the first time. The experiments were performed on SiC structures with natural superlattice under electrical pumping. In combination with earlier reported high-field transport data [17,18,22-24] demonstrating the evolution of the fundamental stages of WSL in SiC natural superlattices, the presented results constitute substantial proof of the pronounced Wannier-Stark localization effect in solid-state objects. It is necessary to add that SiC with natural superlattices opens new possibilities for perspective research in the area of high-field transport phenomena. The discovered intensive THz electroluminescence with a variable frequency can find practical applications for electrically tunable THz emitters. The considerable variation of the emission frequency from 0.3 to 3 THz can be attained through the choice of SiC polytype with appropriate parameters for the superlattice: the highest emission frequency can be achieved with 4H-SiC, but for the lowest emission frequency, any polytype can be chosen from the row of 15R-SiC, 21R-SiC, 27R-SiC, 33R-SiC, and so on.

## Competing interests

The authors declare that they have no competing interests.

## Authors’ contributions

VS conceived, designed, and coordinated the study, determined the directions of investigations, and wrote the manuscript. AA determined the technology of THz optical investigations and wrote the manuscript. AP carried out the theoretical analysis of THz transition physics in Bloch oscillation regime and wrote the manuscript. AZ carried out the optical measurements. AL carried out the electrical measurements of the experimental structures. PS carried out all cycles of electrical contact fabrication to experimental structures. All authors read and approved the final manuscript.

## References

[B1] Bloch F Quantum mechanics of electrons in crystal lattices Z Phys 1928 52 555 600

[B2] Zener C A theory of the electrical breakdown of solid dielectrics Proceedings of the Royal Society of London. Series A 1934 145 855 523 529 10.1098/rspa.1934.0116

[B3] Wannier GH Wave functions and effective Hamiltonian for Bloch electrons in an electric field Phys Rev 1960 117 2 432 439 10.1103/PhysRev.117.432

[B4] Esaki L Tsu R Superlattice and negative differential conductivity in semiconductors IBM J Res and Dev 1970 14 61 65

[B5] Bleuse J Bastard G Voisin P Electric-field-induced localization and oscillatory electro-optical properties of semiconductor superlattices Phys Rev Lett 1988 60 3 220 223 10.1103/PhysRevLett.60.220 10038479

[B6] Mendez EE Agulló-Rueda F Hong JM Stark localization in GaAs-GaAlAs superlattices under an electric field Phys Rev Lett 1988 60 2426 2429 10.1103/PhysRevLett.60.2426 10038348

[B7] Feldmann J Leo K Shah J Miller DAB Cunningham JE Meier T von Plessen G Schulze A Thomas P Schmitt-Rink S Optical investigation of Bloch oscillations in a semiconductor superlattice Phys Rev B 1992 46 7252 7255 10.1103/PhysRevB.46.7252 10002446

[B8] Leo K Bolivar PH Brüggemann F Schwedler R Köhler K Observation of Bloch oscillations in a semiconductor superlattice Solid State Commun 1992 84 10 943 946 10.1016/0038-1098(92)90798-E

[B9] Cho GC Dekorsy T Bakker HJ Kurz H Kohl A Opitz B Bloch oscillations in In-Ga-As-P/In-Ga-As-P heterostructures observed with time-resolved transmission spectroscopy Phys Rev B 1996 54 4420 4423 10.1103/physrevb.54.4420 9986386

[B10] Lyssenko VG Löser F Hasche T Leo K Dignam MM Köhler K Valušis G Direct measurement of the spatial displacement of Bloch-oscillating electrons in semiconductor superlattices Phys Rev Lett 1997 79 301 304 10.1103/PhysRevLett.79.301

[B11] Waschke C Roskos HG Schwedler R Leo K Kurz H Köhler K Coherent submillimeter-wave emission from Bloch oscillations in a semiconductor superlattice Phys Rev Lett 1993 70 3319 3322 10.1103/PhysRevLett.70.3319 10053838

[B12] Shimada Y Hirakawa K Odnoblioudov M Chao KA Terahertz conductivity and possible Bloch gain in semiconductor superlattices Phys Rev Lett 2003 90 046806 12570445 10.1103/PhysRevLett.90.046806

[B13] Leo K Interband optical investigation of Bloch oscillations in semiconductor superlattices Semicond Sci and Technol 1998 13 3 249 263 10.1088/0268-1242/13/3/003

[B14] Verma AR Krishna P Polymorphism and Polytypism in Crystals 1966 Wiley, New York

[B15] Choyke W Patrick L Exciton recombination radiation and phonon spectrum of 6H SiC 6H-SiC Phys Rev 1962 127 1868 1877 10.1103/PhysRev.127.1868

[B16] Jones H The Theory of Brillouin Zones and Electronic States in Crystals 1960 North-Holland Publishing Company, Amsterdam

[B17] Sankin V Naumov A The Wannier-Stark effect and N-shape volt-ampere characteristics in a superlattice of 6H-silicon carbide Superlattices and Microstruct 1991 10 3 353 356 10.1016/0749-6036(91)90340-W

[B18] Sankin V Stolichnov I Negative differential conduction in the Bloch oscillations regime in the hexagonal silicon carbide polytypes 4H, 6H and 8H Superlattices and Microstruct 1998 23 5 999 1004 10.1006/spmi.1996.0571

[B19] Sibille A Palmier JF Wang H Mollot F Observation of Esaki-Tsu negative differential velocity in GaAs/AlAs superlattices Phys Rev Lett 1990 64 52 55 10.1103/PhysRevLett.64.52 10041271

[B20] Beltram F Capasso F Sivco DL Hutchinson AL Chu SNG Cho AY Scattering-controlled transmission resonances and negative differential conductance by field-induced localization in superlattices Phys Rev Lett 1990 64 3167 3170 10.1103/PhysRevLett.64.3167 10041915

[B21] Grenzer J Ignatov AA Schomburg E Renk KF Pavel’ev DG Koschurinov Y Melzer B Ivanov S Schaposchnikov S Kop’ev PS Microwave oscillator based on Bloch oscillations of electrons in a semiconductor superlattice Ann der Phys 1995 507 3 184 190 10.1002/andp.19955070304

[B22] Sankin V Strong field electron transport in silicon carbide superlattice Superlattices and Microstruct 1995 18 4 309 10.1006/spmi.1995.1116

[B23] Sankin V Wannier-Stark localization in the natural superlattice of silicon carbide polytypes Semiconductors 2002 36 717 739 [ doi:10.1134/1.1493739] 10.1134/1.1493739

[B24] Sankin V Lepneva A Electron saturated vertical velocities in silicon carbide polytypes Mater Sciency Forum 2000 338-342 769 772

[B25] Bouchard AM Luban M Bloch oscillations and other dynamical phenomena of electrons in semiconductor superlattices Phys Rev B: Condens Matter and Mater Phys 1995 52 7 5105 5123 10.1103/PhysRevB.52.5105 9981694

[B26] Maksimov A Mal’tsev A Yushin N Nikitina I Sublimation epitaxy of 6H and 4H-SiC on one-inch silicon carbide single-crystal substrates obtained from bulk ingots Tech Phys Lett 1995 21 301 303

[B27] Sankin V Shkrebii P Savkina N Kuznetsov N New Wannier-Stark localization effects in natural 6H-SiC superlattice JETP Lett 2003 77 34 38 [ doi:10.1134/1.1561978] 10.1134/1.1561978

[B28] Andrianov A Zakhar’in A Yassievich I Zinov’ev N Terahertz electroluminescence under conditions of shallow acceptor breakdown in germanium JETP Lett 2004 79 365 367 [ doi:10.1134/1.1772432] 10.1134/1.1772432

